# Prestimulus EEG Power Predicts Conscious Awareness But Not Objective Visual Performance

**DOI:** 10.1523/ENEURO.0182-17.2017

**Published:** 2017-12-12

**Authors:** Christopher S. Y. Benwell, Chiara F. Tagliabue, Domenica Veniero, Roberto Cecere, Silvia Savazzi, Gregor Thut

**Affiliations:** 1 Centre for Cognitive Neuroimaging, Institute of Neuroscience and Psychology, University of Glasgow, Glasgow G12 8QB, United Kingdom; 2 CIMEC – Center for Mind/Brain Sciences, Università degli Studi di Trento, Rovereto 38068, Italy; 3 Perception and Awareness (PandA) Laboratory, Department of Neuroscience, Biomedicine and Movement Sciences, University of Verona, Verona 8 I-37134, Italy

**Keywords:** α, attention, consciousness, EEG, oscillations

## Abstract

Prestimulus oscillatory neural activity has been linked to perceptual outcomes during performance of psychophysical detection and discrimination tasks. Specifically, the power and phase of low frequency oscillations have been found to predict whether an upcoming weak visual target will be detected or not. However, the mechanisms by which baseline oscillatory activity influences perception remain unclear. Recent studies suggest that the frequently reported negative relationship between α power and stimulus detection may be explained by changes in detection criterion (i.e., increased target present responses regardless of whether the target was present/absent) driven by the state of neural excitability, rather than changes in visual sensitivity (i.e., more veridical percepts). Here, we recorded EEG while human participants performed a luminance discrimination task on perithreshold stimuli in combination with single-trial ratings of perceptual awareness. Our aim was to investigate whether the power and/or phase of prestimulus oscillatory activity predict discrimination accuracy and/or perceptual awareness on a trial-by-trial basis. Prestimulus power (3–28 Hz) was inversely related to perceptual awareness ratings (i.e., higher ratings in states of low prestimulus power/high excitability) but did not predict discrimination accuracy. In contrast, prestimulus oscillatory phase did not predict awareness ratings or accuracy in any frequency band. These results provide evidence that prestimulus α power influences the level of subjective awareness of threshold visual stimuli but does not influence visual sensitivity when a decision has to be made regarding stimulus features. Hence, we find a clear dissociation between the influence of ongoing neural activity on conscious awareness and objective performance.

## Significance Statement

Previous research suggests that both the power and phase of neural oscillations occurring immediately before the appearance of a visual stimulus can predict perception of the stimulus. We investigated whether these neural signatures were primarily influencing subjective and/or objective aspects of visual performance. We provide evidence that prestimulus power (∼3–28 Hz) predicts the level of subjective awareness of the stimulus but not whether someone will be more accurate in their ability to discern task-relevant stimulus features. In contrast to previous studies, we found no effect of oscillatory phase on either subjective or objective measures of visual performance. We conclude that prestimulus oscillatory power predicts subjective but not objective measures of visual task performance.

## Introduction

Across multiple presentations of identical stimuli, behavioral responses on psychophysical tasks often vary within the same observer. Intrinsic fluctuations in neural excitability before stimulus presentation provide a possible explanation for this variability of perceptual outcome. Recent EEG/MEG studies employing prestimulus oscillatory activity in specific frequency bands as an index of neural excitability states have shown that prestimulus power (Ergenoglu et al., 2004; Babiloni et al., 2006; Capilla et al., 2014; Limbach and Corballis, 2016; Iemi et al., 2017) and/or phase (Busch et al., 2009; Mathewson et al., 2009; VanRullen et al., 2011) predict the perceptual fate of an upcoming stimulus. One consistent finding is that oscillatory power in the α-band (∼8–14 Hz) immediately preceding stimulus onset negatively correlates with the likelihood of detecting perithreshold visual stimuli (Limbach and Corballis, 2016; Iemi et al., 2017). Additionally, there is evidence linking the alignment of prestimulus oscillatory phase relative to stimulus onset with the likelihood of stimulus detection (Busch et al., 2009; Mathewson et al., 2011; but see Brüers and VanRullen, 2017).

More recently, studies have begun to employ psychophysical modeling techniques, broadly within a signal detection theory (SDT) framework (Green and Swets, 1966), to investigate the mechanism by which prestimulus activity influences perception (Chaumon and Busch, 2014; Limbach and Corballis, 2016; Iemi et al., 2017). These studies have provided converging evidence that prestimulus α power may primarily bias perception by influencing the decision criterion, and also subsequent decision confidence (Samaha et al., 2017), rather than influencing perceptual sensitivity (Lange et al., 2013; Chaumon and Busch, 2014; Limbach and Corballis, 2016; Sherman et al., 2016; Craddock et al., 2017; Iemi et al., 2017; Samaha et al., 2017). Iemi et al. (2017) proposed that in states of low α power (indexing high cortical excitability), participants are more likely to report detection (both hits and false alarms) than in states of high α power (low excitability). In contrast, discrimination measures, requiring evaluation of some veridical characteristic of the stimulus, are unaffected by α power, since both decision-related “signal” and “noise” are equally affected by baseline excitability. Accordingly, Samaha et al. (2017) found that prestimulus α power was negatively correlated with decision confidence in a 2-alternative forced choice (2-AFC) orientation discrimination task, but was not correlated with decision accuracy. These recent findings appear somewhat at odds with previous studies which have found a positive correlation between prestimulus α power and visual detection performance (Linkenkaer-Hansen et al., 2004; Babiloni et al., 2006; Mayer et al., 2016).

While the studies described above suggest that prestimulus α power predicts decision criterion and subsequent confidence rather than objective performance, it remains unclear at which level of processing these effects occur. For instance, criterion and confidence may be influenced primarily at the nonsensory levels of decision strategy and metacognition. Additionally, both measures are likely to be related to the level of subjective awareness (i.e., subjective visibility) of the stimulus, and hence α power may primarily influence perceived stimulus visibility, which in turns affects the decision criterion and confidence. In the current study, we sought to investigate to what extent oscillatory activity before stimulus onset is linked to subjective awareness. We employed a 2-AFC luminance discrimination task using three perithreshold stimulus intensities, and catch trials in which no stimulus was presented, in combination with single-trial ratings of subjective awareness (Ramsøy and Overgaard, 2004; Tagliabue et al., 2016). The inclusion of graded stimulus intensities and catch trials allowed us to test whether the relationship between prestimulus α power and perceptual reports is uniform regardless of the presence and intensity of the stimulus (in line with a decision criterion effect) or rather depends on there being a stimulus present and how strong it is (in line with a perceptual response gain effect; Chaumon and Busch, 2014).

Hence, based on the evidence outlined above, we hypothesized that prestimulus α power would predict subjective awareness ratings but not discrimination accuracy. By grading stimulus intensity and including catch trials, we tested to what extent the influence of prestimulus α on the decision criterion/perceptual bias (Iemi et al., 2017) and decision confidence (Samaha et al., 2017), in the absence of any influence on perceptual sensitivity, may be parsimoniously explained by an influence of α power on the level of conscious awareness of the visual percept. Additionally, given that the mechanism by which prestimulus phase influences perception remains largely unknown (for theories, see VanRullen and Koch, 2003; Jensen et al., 2012; VanRullen, 2016a), we also sought to establish whether prestimulus phase predicts subjective awareness ratings and/or discrimination accuracy.

## Materials and Methods

### Participants

A total of 14 participants (seven females, two left-handed, mean age ± SD: 23.79 ± 3.17) were recruited for the study. All reported normal or corrected-to-normal vision and no history of neurological or psychiatric disorders. They all gave their written informed consent to participate in the study. The study was approved by the Ethics Committee of the College of Science and Engineering at the University of Glasgow and conducted in accordance with the 2013 Declaration of Helsinki.

### Experimental procedure

The experiment consisted of two sessions performed on two consecutive days. The first session involved a threshold assessment (see Threshold titration below) and familiarization of the participants with the stimuli. During the second session, after a threshold reassessment, participants performed a forced choice discrimination task while EEG was simultaneously recorded (see EEG session below).

### Stimuli

Each participant was presented with Gaussian patches, which were parametrically varied and individually adjusted in luminance (i.e., manipulated in saliency). Half of the stimuli were lighter and the other half darker than the background for use in the lighter-darker discrimination task during the EEG experiment.

The stimuli were black or white circular patches with a Gaussian envelope (size = 1.3°), presented on a gray background (RGB: 127, 127, 127) in the upper right visual field (5° of vertical and 10° of horizontal eccentricity from the fixation cross). Before the experimental task, the contrast (i.e., transparency) of the black and white Gaussian patches was individually adjusted to obtain perithreshold stimuli of different luminance that appeared as light and dark gray, respectively. Specifically, six stimulus luminance levels (three lighter and three darker than the gray background) were identified for each participant by means of a threshold assessment procedure (see next paragraph for further details).

### Threshold titration

For the threshold titration session, participants sat with their head on a chin rest in front of a CRT monitor (resolution 1280 × 1024, refresh rate of 100 Hz) at a viewing distance of 57 cm. The aim of the titration session was to identify six contrast values (yielding six luminance levels: three light and three dark patches) corresponding to 25%, 50%, and 75% of correct detection performance. The thresholds were measured using the method of constant stimuli. At the beginning of the session (first day), ten evenly spaced contrast values ranging from 0.025% to 0.116% of the maximal contrast of the black/white patches were presented in a randomized order in the right visual field (for details, see Stimuli above). This first phase included two blocks: on each block, all contrast values were tested seven times together with 14 stimulus-absent trials (catch trials), resulting in a total number of 308 trials per participant. On each trial, a warning tone (1000 Hz, 150 ms) preceded the stimulus with a 1000-ms cue-stimulus interval. Participants were asked to keep their eyes on a central fixation cross and press the spacebar whenever they perceived a stimulus, and to withhold responses when not perceived (1 s time limit for response). At the end of the two blocks, a sigmoid function was fit to the data of both light and dark stimulus trials separately and contrast values yielding detection thresholds of 25%, 35%, 50%, 65%, and 75% were extracted for each participant. These contrast levels were then tested again in two blocks, including 10 trials for each contrast/stimulus type (light and dark stimuli) and 14 catch trials, resulting in a total number of 228 trials per participant.

On the second day of testing and before EEG recording, a short threshold reassessment was performed to verify that participants’ performance was comparable to that obtained in the first session. To this end, the contrast values previously identified (5 for light and 5 for dark patches) plus those corresponding to 0% and 100% detection accuracy were each presented seven times together with 14 catch trials (total of 182 trials). If luminance values resulting in detection thresholds of ∼25%, 50%, and 75% were confirmed, they were selected for the behavioral task during the EEG recording. If not confirmed, a sigmoid function was once again fit to the data and new contrast levels were extracted and retested with the same procedure. The threshold reassessment procedure had to be repeated for four subjects.

### Discrimination task and EEG experiment

**Figure 1.**
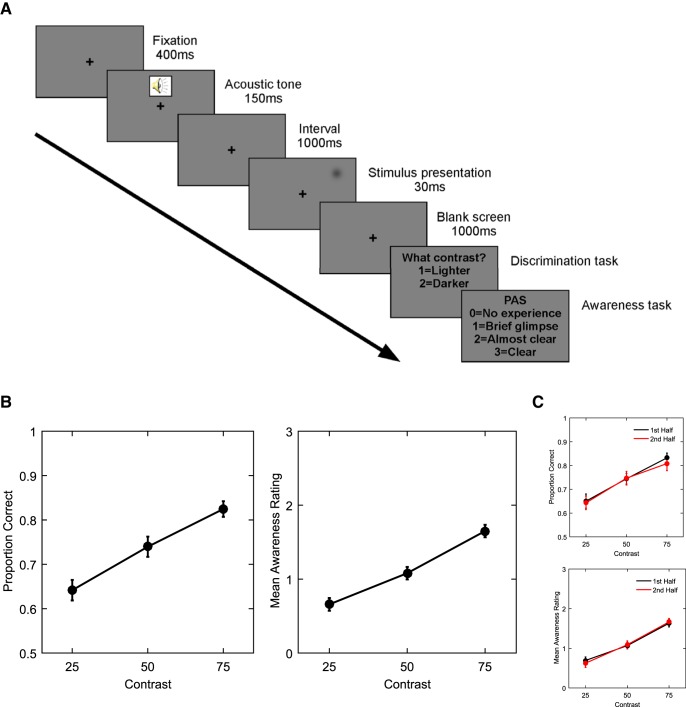
Task design and performance. ***A***, Each trial began with a black fixation cross for 400 ms followed by a 1000-Hz warning tone (150 ms). After a 1000-ms interval, a light or dark Gaussian patch was presented for 30 ms (three frames) in the right visual field. A 1000-ms blank screen (fixation cross present) was then followed by a response prompt asking the participants to judge the brightness of the stimulus as compared with the gray background, indicating either lighter or darker. After the response, another prompt asked participants to rate the quality of their perception on the four-point PAS. ***B***, Group-averaged proportion of correct responses (left) and mean awareness ratings (right) as a function of stimulus contrast (25%, 50%, and 75% of detection threshold). ***C***, Group-averaged proportion of correct responses (top) and mean awareness ratings (bottom) as a function of stimulus contrast (25%, 50%, and 75% of detection threshold) and time-on-task (first half of experiment = black lines; second half = red lines). Both accuracy and awareness rating linearly increased from low to high stimulus contrast and these effects were similar in both the first and second halves of the experiment. All error bars indicate within-subject ± SEM.

During the EEG session, participants performed a 2-AFC luminance discrimination task. Each trial (Fig. 1*A*) started with a black fixation cross for 400 ms, followed by a 1000-Hz warning tone (150 ms). After a 1000-ms interval, a light or dark Gaussian patch (whose luminance levels were determined during the threshold assessment) was presented for 30 ms (three frames) in the upper right visual field. A 1000-ms screen with only the fixation cross was then displayed, followed by a response prompt asking the participants to judge the brightness of the stimulus relative to the gray background by pressing a button (“1” key on numeric pad of keyboard) for “lighter” and another button (“2” key on numeric pad of keyboard) for “darker” using their right index and middle fingers, respectively. The participants were required to guess in trials in which they did not see any stimulus. After the button press, another response prompt asked participants to rate the quality of their perception on the four-point perceptual awareness scale (PAS; Ramsøy and Overgaard, 2004). The four PAS categories were: (0) no experience of the stimulus, (1) a brief glimpse, (2) an almost clear experience, and (3) a clear experience. Responses were given by pressing four different buttons on the keyboard (“0,” “1,” “2,” and “3” on the numeric pad). Participants were instructed that these categories index the clarity of the visual experience, regardless of whether they thought their discrimination decision was correct or not. Although awareness and confidence are likely to be highly correlated, Sandberg et al. (2010) showed that instructing participants not to use the PAS scale as a proxy for confidence resulted in marked differences in the use of the scale compared to a four-point confidence scale, with the PAS scale leading to more graded, and hence exhaustive, responses.

The experimental session was divided into 10 blocks. Each block consisted of 80 trials: 10 trials for each individually adjusted stimulus contrast (25%, 50%, and 75% of detection threshold) and stimulus type (light and dark), together with 20 catch trials, thus yielding a total of 800 trials across blocks. The order of the trials within each block was fully randomized. Both the threshold assessment and the actual behavioral task were programmed and run in MATLAB (MathWorks Inc.), using the Psychophysics Toolbox (Brainard, 1997; Pelli, 1997).

### Behavioral analysis

To evaluate the effectiveness of the experimental manipulations, statistical analyses were conducted separately for the discrimination accuracy scores and awareness ratings. The dependent measures were the proportion of correct responses (discrimination accuracy) and mean PAS rating (indexing visual awareness). The independent variables were the stimulus contrast (25%, 50%, and 75% of detection threshold) and the time within the experimental session (first half of trials vs second half). The time factor was introduced to test for nonstationarity in the psychophysical responses over time, which may provide an alternative explanation for apparent trial-by-trial correlations between EEG and perceptual measures (Benwell et al., 2013, 2017; Bompas et al., 2015). Hence, three (contrast: 25%, 50%, 75%) × two (time: first half, second half) repeated measures ANOVAs were performed on both the accuracy and rating measures separately. Effect sizes were also estimated using partial η^2^ and Cohen’s *d*.

### EEG recording and analysis

Continuous EEG was recorded with two BrainAmp MR Plus units (Brain Products GmbH) at a sampling rate of 1000 Hz through 61 Ag/AgCl pellet pin scalp electrodes placed according to the 10-10 International System. Two extra electrodes served as ground (TP9) and on-line reference (AFz). Electrode impedances were kept below 10 kΩ. All scalp channels were rereferenced off-line to the average of all electrodes. Preprocessing steps were performed using Brain Vision Analyzer 2.0 (Brain Products). Offline, continuous data were filtered for power line noise using a 2-Hz notch filter centred at 50 Hz. Additional low (85 Hz) and high-pass (0.1 Hz) filters were applied using a zero-phase second-order Butterworth filter. Independent component analysis (ICA; Bell and Sejnowski, 1995) was applied to identify and remove eye blinks and muscle artifacts. The data were segmented into epochs of 5 s starting −2.5 s before stimulus onset and then down sampled to 250 Hz. All epochs were then visually inspected and removed if contaminated by residual eye movements, blinks, strong muscle activity, or excessive noise. On average, ∼5% of the trials were discarded per participant due to artifacts.

Fourier-based spectro-temporal decomposition of the artifact-free data were performed using the ft_freqanalysis function (wavelet convolution method: “mtmconvol”) from the FieldTrip toolbox (Oostenveld et al., 2011), yielding complex-valued time-frequency planes. A temporal resolution was maintained by decomposing overlapping 0.5-s segments of trial time series, consecutively shifted forward in time by 0.02 s. Data segments were multiplied with a Hanning taper and then zero-padded to a length of 1 s to achieve a frequency resolution of 1 Hz across the range of 3 to 40 Hz. The data were then reepoched from −1 to 1 s relative to stimulus onset. We sought to investigate spectral EEG predictors of both discrimination accuracy and visual awareness ratings. The two spectral measures investigated were power and phase.

#### EEG power analysis

Single-trial power was obtained for all time-frequency points as follows:

$${EEGpower(t,f)=|F(t,f)|}^{2}$$

where *F* is the complex Fourier coefficient corresponding to time window *t* and frequency *f.* The absolute power values were additionally normalized using a decibel (dB) transformation (Matlab pow2db function).

The relationships between single-trial power and both discrimination accuracy and visual awareness ratings were first tested using a multiple regression approach (inspired by Samaha et al., 2017 ). At the single participant level, for each electrode, frequency and time point, regression coefficients were estimated for a model with single-trial accuracy and awareness ratings (and their interaction) as predictors of single-trial EEG power:

$$EEG=a+b_{acc}*Acc+b_{rate}*Rate+b_{int}*Acc*Rate+\varepsilon$$

where *EEG* is the single-trial power estimates, Acc is a column of values corresponding to single-trial discrimination accuracy [dummy coded as 0 (incorrect) and 1 (correct)] and Rate is a column of single-trial PAS ratings (0:3). The regression coefficient $b_{int}$represents the direction and strength of the interaction term, indexing whether the relationship between one predictor variable and the EEG power depends on the level of the other predictor variable. a is the model intercept and ε the error term. Although we did not hypothesize any interaction between accuracy and awareness ratings in terms of their relationship with EEG power, we included the interaction term initially to make sure the model was being specified correctly. In the absence of a significant interaction term, the independent contributions to the prediction of EEG power of awareness and accuracy (main effects) can be estimated in a linear model with no interaction term:

$$EEG=a+b_{acc}*Acc+b_{rate}*Rate+\varepsilon$$

where $b_{acc}$ and $b_{rate}$ represent the independent contributions to the prediction of EEG power of both discrimination accuracy and PAS ratings, respectively.

Additionally, to ensure that the results were not dependent on our choice of predictor and outcome variables (i.e., behavior predicting EEG power) and also to control for any potential influence of multicollinearity in the multiple regression model described above, we also implemented two separate models in which EEG power was entered as the predictor and the behavioral measure as the outcome variable. For the PAS ratings, coefficients were estimated for the following linear model:

$$Rate=a+b_{EEG}*EEG+\varepsilon$$

where $b_{EEG}$ indexes the direction and strength of the relationship between single-trial EEG power and PAS ratings. For discrimination accuracy and given the binary nature of this variable, a logistic regression was performed according to the following formula:

$$log(\frac{P\left(Corr\right)}{1-P(Corr)})=a+b_{EEG}*EEG$$

where $b_{EEG}$ indexes the direction and strength of the relationship between single-trial EEG power and the probability of being correct *(P(Corr))*.

At the group level, regression coefficients were combined across participants for statistical analysis. More specifically, if at a given data point (electrode/frequency/time), EEG power systematically covaries linearly with the perceptual measure (discrimination accuracy or awareness rating) then regression slopes should show a consistent directionality across participants. Alternatively, if there is no systematic linear relationship between EEG power and the perceptual measure, then regression slopes across participants should be random (centered around 0). Hence, for each EEG/behavior relationship we performed one-sample *t* tests (test against 0) on the regression coefficient values across participants at all data points (i.e., all electrodes, frequencies, time points). To control the familywise error rate (FWER) across the large number of comparisons, cluster-based permutation testing (Maris and Oostenveld, 2007) was employed. Calculation of the test statistic involved the following: based on the initial one-sample *t* tests, all *t* values above a threshold corresponding to an uncorrected *p* value of 0.05 were formed into clusters by grouping together adjacent significant time-frequency points and electrodes. This step was performed separately for samples with positive and negative *t* values (two-tailed test). Note that for a significant sample to be included in a cluster, it was required to have at least 1 adjacent neighboring significant sample. The spatial neighborhood of each electrode was defined as all electrodes within ∼5 cm, resulting in a mean of 6.3 (minimum = 3, maximum = 8) and median of 7 neighbors per electrode. The *t* values within each cluster were then summed to produce a cluster-level *t* score (cluster statistic). Subsequently, this procedure was repeated across 2000 permutations of the data (condition labels were shuffled for a random subset of participants on each iteration) with the most extreme cluster-level *t* score on each iteration being retained to build a data driven null hypothesis distribution. The location of the original real cluster-level *t* scores within this null hypothesis distribution indicates how probable such an observation would be if the null hypothesis were true (no systematic difference from 0 in regression slopes across participants). Hence, if a given negative/positive cluster had a cluster-level *t* score lower/higher than 97.5% of the respective null distribution *t* scores, then this was considered a significant effect (5% α level).

#### Follow-up EEG power analysis

To further investigate the nature of any detected relationships between prestimulus power and behavior, and to confirm the results of the single-trial regression analysis, we performed an additional analysis on the data from electrode-time-frequency points included in any significant clusters before stimulus onset. Single-trial, cluster-averaged, prestimulus power values were extracted for each participant and trials were split into “above” and “below” median power bins. The proportion of correct responses and mean PAS ratings were then calculated per prestimulus power bin (above and below median) separately for each luminance/contrast combination, i.e., for luminance increments (stimuli lighter than background) and decrements (stimuli darker than background), respectively, at the 25%, 50%, and 75% contrast levels. Subsequently, repeated measures ANOVAs with the factors prestimulus cluster power (high, low), contrast (25%, 50%, 75%) and luminance (darker, lighter) were performed on both the accuracy and awareness rating measures separately.

This additional analysis was performed for several reasons. First, the multiple regression approach is potentially confounded by multicollinearity because the accuracy and visual awareness measures are likely correlated. Hence, it may be problematic to detect independent contributions from each predictor in a regression model. If real effects are masked by our choice of regression model, then we would expect them to be apparent in the ANOVAs performed separately for accuracy and visual awareness. Second, the initial regression analysis does not account for differences in stimulus luminance (i.e., darker or lighter than background) or contrast (i.e., stimulus intensity: 25%, 50%, or 75%). Because a novel aspect of our design was that the stimuli on any given trial could either be luminance increments or decrements (relative to the background), it is of interest to test whether prestimulus oscillations influence perception in the same way for both stimulus types. Third, the level of stimulus intensity (as indexed by the contrast) may interact with any effect of prestimulus activity on perception. For example, Chaumon and Busch (2014) presented a range of stimulus intensities in a visual detection task and found that high prestimulus α power only reduced detection of stimuli of the highest intensities tested, in line with a reduction of response gain rather than a change in sensitivity.

#### Catch trial power analysis

Recent studies by Limbach and Corballis (2016) and Iemi et al. (2017) have found that in states of low relative to high α power, the false alarm rate is increased along with the hit rate. We also tested for this in the current data by performing a separate regression analysis on the catch trials only. A mean number of 188 catch trials (minimum = 153, maximum = 200) per participant were entered into the analysis. For each electrode, frequency and time point in each participant, regression coefficients that describe the relationship between single-trial power and PAS ratings on the catch trials were estimated according to the linear model:

$$EEG=a+b_{catchrate}*CatchRate+\varepsilon$$

where *EEG* is the single-trial power estimates and CatchRate is a column of single-trial PAS ratings (0:3). The regression coefficient $b_{catchrate}$ was our measure of interest. a is the model intercept and ε the error term. The same group-level cluster-based permutation analysis was then employed as for the accuracy and awareness analyses from the noncatch trials described above.

Finally, in analogy to the follow-up power analysis above, we also specifically investigated the relationship between catch trial power and PAS ratings from prestimulus clusters identified in the noncatch trial analyses. Here, we calculated the mean PAS ratings for catch trials from high and low power trial bins (averaged over the data points from the significant cluster) in each participant and the difference was tested using a paired-samples *t* test.

#### Bayes factor (BF) analysis of EEG power results

To directly estimate evidence for both the null hypothesis (no relationship between EEG power and behavioral measure) and alternative hypothesis (significant relationship between EEG power and behavioral measure), we also performed BF analyses using the data points from the detected significant cluster (see Iemi et al., 2017 for a similar approach). This analysis allowed us to test whether the absence of a significant relationship between EEG power and accuracy was likely to be due to a lack of statistical power or rather because the null hypothesis was likely to be true. A BF below 1/3 indicates evidence for the null hypothesis, above 3 indicates evidence for the alternative hypothesis and between 1/3 and 3 indicates that the evidence is inconclusive (potentially due to a lack of statistical power; Rouder et al., 2009). For all data points included in a significant prestimulus EEG power/awareness cluster (detected in the regression analysis), the BF was calculated with a prior which followed a Cauchy distribution with a scale factor of 0.707 (Rouder et al., 2009). For each time point, the percentage of electrode-frequency points showing evidence for the null and alternative hypotheses, respectively, were calculated. This analysis was performed separately for both the awareness and accuracy data.

#### Phase analysis

To investigate whether single-trial discrimination accuracy and/or subjective awareness ratings depend on the phase of ongoing oscillatory activity before stimulus onset, we employed a phase opposition analysis (VanRullen, 2016b). Essentially, this analysis tests whether trials associated with one perceptual outcome (i.e., correct discrimination or high subjective awareness) differ in terms of their distribution of oscillatory phases for a given time-frequency point compared to trials associated with the opposite perceptual outcome (i.e., incorrect discrimination or low subjective awareness). The analysis involves a comparison of intertrial phase coherence (ITPC) measured over all trials (serving as a baseline) with ITPC measured separately for the trial group from each condition (i.e., correct vs incorrect discrimination and high vs low subjective awareness). If the ITPC from each condition is larger than the total ITPC then this suggests that the two conditions are phase-locked to different phase angles. ITPC was calculated as follows:

$$ITPC\left(t,f\right)=|\frac{1}{n}\sum_{k=1}^{n}\frac{F_{k}\left(t,f\right)}{\left|F_{k}\left(t,f\right)\right|}|$$

where *F* is the complex Fourier coefficient corresponding to time window *t* and frequency *f*, *n* is the number of trials and *k* is the individual trial index. The ITPC was calculated in this way over all trials and separately for those trials corresponding to correct discrimination, incorrect discrimination, high awareness ratings (2 and 3 PAS ratings) and low awareness ratings (0 and 1 PAS ratings), respectively.

We employed the phase opposition sum (POS) method (VanRullen, 2016b) to test for differences in preferred phase angle between “correct” and “incorrect” trials (for the discrimination accuracy analysis) and “high awareness rating” and “low awareness rating” trials (for the visual awareness analysis), respectively. The POS is calculated as follows:

$$POS={ITPC}_{A}+{ITPC}_{B}-2*{ITPC}_{ALL}$$

where ${ITPC}_{A}$ and ${ITPC}_{B}$ are the ITPC calculated separately for the two trial-types to be compared (i.e., correct vs incorrect response trials or high vs low awareness rating trials) and ${ITPC}_{ALL}$ is the ITPC calculated across all trials regardless of condition. *POS* will be positive when the ITPC of each trial group exceeds the overall ITPC; the main situation of interest, which indicates significant phase opposition between the two conditions.

Statistical analysis was first performed at the level of individual participants using a permutation test. For each participant, the trial assignment to group A or B was randomly permuted 2000 times and the POS value calculated and stored on each iteration. For each electrode-time-frequency point, the *p* value was calculated as the proportion of permutations that yielded a higher POS than the observed data. Hence, the *p* value reflects the likelihood of observing the actual POS value if the null hypothesis (no phase opposition) was true. The individual participant *p* values were subsequently combined using Fisher’s combined probability test (Fisher, 1925), which yielded a single group-level *p* value for each electrode-time-frequency point. Only prestimulus data points (-1:0 s relative to stimulus onset) were entered into group-level statistical analysis because phase opposition measures are difficult to interpret poststimulus when strong evoked activity (event-related potentials) implies that most trials are phase locked to similar phase angles (VanRullen, 2016b). To control for multiple comparisons, we employed nonparametric false discovery rate (FDR) correction (Benjamini and Yekutieli, 2001) with a threshold (*q* value) of 0.05. The entire analysis was performed separately for discrimination accuracy and visual awareness, respectively.

#### Follow-up EEG phase analysis

Additionally, we performed two control POS analyses. In the first control analysis, trial numbers were matched between the two outcomes. We did this because phase opposition measures lose statistical power when there is an asymmetry in trial numbers between the two conditions (VanRullen, 2016b). Hence, within each participant, we equalized correct and incorrect trials (for the discrimination accuracy analysis) and high awareness rating and low awareness rating trials (for the visual awareness analysis) by randomly selecting from the higher likelihood outcome the same number of trials present for the lower likelihood outcome. This resulted in an average equalized number of trials per outcome across participants of 201 (minimum = 128, maximum = 281) for visual awareness (high awareness rating vs low awareness rating) and 149 (minimum = 68, maximum = 202) for accuracy (correct and incorrect). In the second control analysis, we entered only the stimulus contrast (one of three levels) which had the most equal distribution of outcomes per participant (the most equal contrast was not identical across participants). This analysis was implemented in case the differing saliency of the contrasts employed were to mask phase opposition between outcomes when different contrasts are collapsed (as may be predicted by the model of Jensen et al., 2012). This resulted in an average equalized number of trials per outcome across participants of 81 (minimum = 63, maximum = 95) for visual awareness (high awareness rating vs low awareness rating) and 68 (minimum = 39, maximum = 87) for accuracy (correct vs incorrect).

## Results

### Behavioral results

After the threshold assessment, the mean luminance value chosen corresponded to 0.0427% of the maximal contrast of the light/dark patches for 25% detection performance, 0.0491% for 50% and 0.0575% for 75% (lighter and darker stimuli collapsed together). Figure 1*B* plots the group-averaged proportion of correct responses (left plot) and mean awareness ratings (right plot) as a function of stimulus contrast (25%, 50%, and 75% of detection threshold) and Figure 1*C* plots the same measures (accuracy data = top plot, awareness rating data = bottom plot) as a function of both stimulus contrast and time-on-task (first half of experiment = black lines, second half = red lines) to account for potential effects of time-on-task (Benwell et al., 2017). Both accuracy and awareness ratings linearly increased from low to high stimulus contrast (Fig. 1*B*). These effects were similar in both the first and second halves of the experiment (Fig. 1*C*).

The repeated measures ANOVA on the proportion of correct responses (discrimination accuracy) revealed a significant main effect of stimulus contrast (*F*_(1,26)_ = 42.244, *p* < 0.001,$\eta_{p}^{2}$ = 0.765, linear contrast: *F*_(1,13)_ = 83.04, *p* < 0.001,$\eta_{p}^{2}$ = 0.865), no effect of time-on-task (*F*_(1,26)_ = 0.343, *p* = 0.568,$\eta_{p}^{2}$ = 0.026) and no stimulus contrast × time-on-task interaction (*F*_(1,26)_ = 0.444, *p* = 0.646,$\eta_{p}^{2}$ = 0.033). Pairwise comparisons employed to analyze the simple effects of contrast revealed increases in the proportion of correct responses from the 25%–50% (*t*_(13)_ = 4.521, *p* = 0.001, Cohen’s *d* = 1.301), the 25%–75% (*t*_(13)_ = 10.496, *p* < 0.001, Cohen’s *d* = 2.806) and the 50%–75% (*t*_(13)_ = 4.955, *p* < 0.001, Cohen’s *d* = 1.564) contrast conditions. Hence, the experimental manipulation of stimulus contrast led to a corresponding increase in discrimination accuracy and this effect was not modulated by time-on-task.

The repeated measures ANOVA on the mean PAS ratings (subjective awareness) revealed a significant main effect of stimulus contrast (*F*_(1,26)_ = 87.803, *p* < 0.001,$\eta_{p}^{2}$ = 0.871, linear contrast: *F*_(1,13)_ = 171.571, *p* < 0.001,$\eta_{p}^{2}$ = 0.93), no effect of time-on-task (*F*_(1,26)_ = 0.001, *p* = 0.973,$\eta_{p}^{2}$ < 0.001) and no stimulus contrast × time-on-task interaction (*F*_(1,26)_ = 1.671, *p* = 0.208,$\eta_{p}^{2}$ = 0.114). Pairwise comparisons were again employed to analyze the simple effects of contrast. These revealed increases in mean PAS rating from the 25%–50% (*t*_(13)_ = 5.577, *p* < 0.001, Cohen’s *d* = 1.573), the 25%–75% (*t*_(13)_ = 13.086, *p* < 0.001, Cohen’s *d* = 3.58) and the 50%–75% (*t*_(13)_ = 7.719, *p* < 0.001, Cohen’s *d* = 2.073) contrast conditions. Hence, the experimental manipulation of stimulus contrast led to a corresponding increase in PAS ratings and this effect was not modulated by time-on-task.

### EEG results

#### Prestimulus power predicts visual awareness ratings but not discrimination accuracy

**Figure 2.**
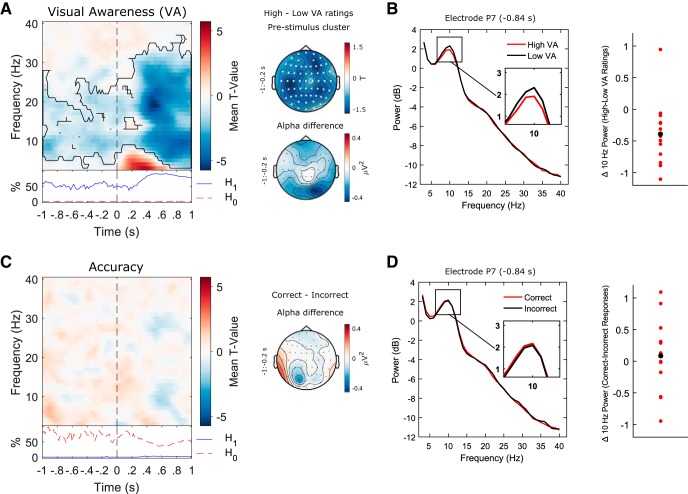
Relationship between oscillatory power and perception. ***A***, The results of a single-trial regression analysis revealed that prestimulus power was negatively correlated with visual awareness ratings (i.e., high power was associated with low PAS ratings and low power with high PAS ratings, black contour denotes significant cluster-corrected effects; *p* < 0.05). Stimulus onset is highlighted by a vertical black dashed line. The bottom inset plots the time course of the percentage of electrode-frequency points within the significant cluster with BFs showing evidence for the null (H_0_: no EEG/awareness relationship; dashed red line) and alternative hypotheses, respectively (H_1_: significant EEG/awareness relationship; solid blue line). As expected, the percentage of data points providing evidence for H_1_ far outnumbered those providing evidence for H_0_. The prestimulus effect was widely distributed over all electrodes as indicated by the topographical representation of the effect (upper right panel; electrodes included in the significant cluster are highlighted in white). The lower right panel plots the scalp topography of the group-average difference in prestimulus α (8–14 Hz) power between high PAS rating (ratings 2 and 3) trials and low PAS rating (ratings 0 and 1) trials. ***B***, Group-averaged frequency spectra computed separately for high PAS rating trials (red lines) and low PAS rating trials (black lines). Compared to low PAS rating trials, high PAS rating trials were associated with decreased prestimulus α power. This effect was highly consistent across participants as shown by the scatterplot (right panel; black dot represents the mean difference value) of the difference in mean 10-Hz power between high and low PAS rating trials for each participant. ***C***, No relationship was found between EEG power and discrimination accuracy in any of the time-frequency ranges examined. The bottom inset plots the time course of the percentage of electrode-frequency points from the significant EEG/awareness cluster with BFs showing evidence for the null (H_0_: no EEG/accuracy relationship; dashed red line) and alternative hypotheses, respectively (H_1_: significant EEG/accuracy relationship; solid blue line). The percentage of data points providing evidence for H_0_ far outnumbers those providing evidence for H_1_. The right panel plots the scalp topography of the difference in prestimulus α power between correct and incorrect trials. ***D***, Group-averaged frequency spectra computed separately for correct (red lines) and incorrect trials (black lines). No difference in power was observed between correct and incorrect trials. The right panel plots the difference in mean 10-Hz power between correct and incorrect trials for each participant (black dot represents the mean difference value).

The initial model tested included both accuracy and PAS ratings along with their interaction term as predictors of EEG power. However, no significant interaction clusters were identified and so the subsequent results are from a model without the interaction term, allowing for estimation of the relationships between EEG power and PAS ratings and accuracy, respectively. Figure 2*A*, left panel, plots *t* values averaged across all 60 electrodes at each time point (from −1 to +1 s poststimulus) denoting the strength of the EEG power, PAS rating relationship, across frequencies of 3–40 Hz. These *t* values represent group-level tests of whether regression coefficients (EEG power vs PAS rating) from the individual single-trial analyses show a systematic linear relationship (i.e., are significantly different from 0) across participants. We found a negative relationship between EEG power and subjective awareness (i.e., low power was associated with high PAS ratings and high power with low PAS ratings) throughout the epoch (cluster statistic = −196751, *p* = 0.0005). In the prestimulus period of interest (−1:0 s relative to stimulus onset), the effect spanned from 3–28 Hz and was widely distributed over all electrodes as indicated by the topographical representation of the effect (data averaged over all time-frequency points included in the cluster from −1 to −0.2 s relative to stimulus onset; Fig. 2*A*, upper right map). Figure 2*A*, lower right map, plots the scalp topography of the difference in α (8-14 Hz) power between high PAS rating trials (ratings 2 and 3) and low PAS rating trials (ratings 0 and 1) during the same prestimulus period (-1:-0.2 s). Figure 2*B*, left panel, plots the group averaged frequency spectra computed separately for high PAS rating trials (red lines) and low PAS rating trials (black lines) from the data point corresponding to the peak *t* value in the prestimulus cluster (electrode P7, −0.84 s). Compared to low PAS rating trials, high PAS rating trials were associated with decreased prestimulus α power. This effect was highly consistent across participants as shown by the scatterplot (Fig. 2*B*, right panel) of the difference in mean 10-Hz power between high and low PAS rating trials for each participant.

In contrast, no relationship was found between EEG power and discrimination accuracy in any of the time-frequency ranges examined [see Fig. 2*C*, left panel, for time-frequency plot, and right panel, for the scalp topography of the difference in α (8-14 Hz) power between correct and incorrect trials during the prestimulus period, i.e., −1:–0.2 s]. Figure 2*D*, left panel, plots the group averaged frequency spectra computed separately for correct (red lines) and incorrect trials (black lines) from the data point corresponding to the peak *t* value in the visual awareness analysis (electrode P7, −0.84 s). No difference in power was observed between correct and incorrect trials (see also the scatterplot in Fig. 2*D* of the difference in mean 10-Hz power between correct and incorrect trials for each participant).

To provide direct evidence as to whether the lack of an effect in the accuracy analysis truly reflected a null result or rather data insensitivity, an additional Bayesian analysis was performed. Here, we assumed that any effect of EEG on accuracy is likely to coincide in electrode-time-frequency space with the effect of EEG on visual awareness. Therefore, for those electrode-frequency points included in the significant negative cluster from the awareness analysis, we calculated at each time point the percentage showing evidence for H_0_ (no EEG/behavior relationship) and H_1_ (EEG/behavior relationship), respectively. As expected, for the awareness analysis (from which the cluster was derived), the percentage of data points providing evidence for H_1_ far outnumbered those providing evidence for H_0_ (Fig. 2*A*, left panel, bottom inset). However, the reverse was true for the accuracy analysis where the percentage of data points providing evidence for H_0_ now far outnumbered those providing evidence for H_1_ (Fig. 2*C*, left panel, bottom inset). This analysis provided strong evidence that prestimulus EEG power had no effect on discrimination accuracy.

**Figure 3.**
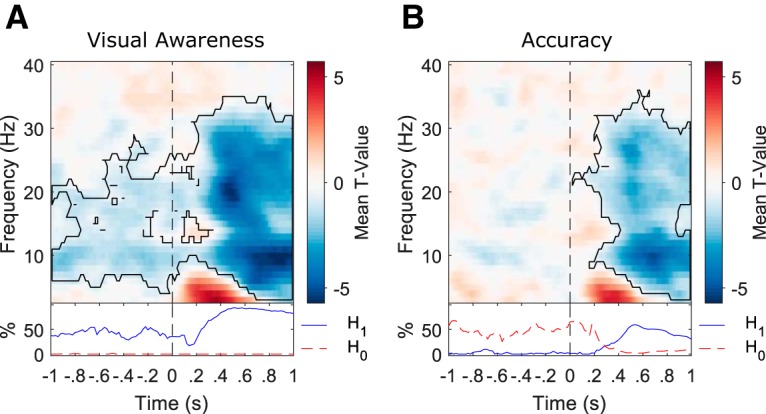
Confirmation of the relationship between oscillatory power and perception from separate regression models with EEG predicting behavior. ***A***, A single-trial linear regression analysis with EEG as a predictor of visual awareness ratings confirmed the negative relationship (i.e., high power was associated with low PAS ratings and low power with high PAS ratings, black contour denotes significant cluster-corrected effects; *p* ≤ 0.05). The bottom inset plots the time course of the percentage of electrode-frequency points within the significant cluster with BFs showing evidence for the null (H_0_: no EEG/awareness relationship; dashed red line) and alternative hypotheses, respectively (H_1_: significant EEG/awareness relationship; solid blue line). The percentage of data points providing evidence for H_1_ far outnumbered those providing evidence for H_0_. ***B***, A single-trial logistic regression analysis with EEG as a predictor of accuracy did not reveal any prestimulus relationship. The bottom inset plots the time course of the percentage of electrode-frequency points from the significant EEG/awareness cluster with BFs showing evidence for the null (H_0_: no EEG/accuracy relationship; dashed red line) and alternative hypotheses, respectively (H_1_: significant EEG/accuracy relationship; solid blue line). The percentage of prestimulus data points providing evidence for H_0_ far outnumbers those providing evidence for H_1_.

These prestimulus results were further supported by additional analyses in which we reversed the predictor and outcome variables in separate regression models for each behavioral measure. When EEG power was entered as the predictor and the behavioral measure as the outcome variable for both PAS ratings (Fig. 3*A*) and accuracy (Fig. 3*B*) separately, a negative relationship was again found between prestimulus power and PAS ratings but not between prestimulus power and accuracy. The negative poststimulus relationship between EEG power and accuracy, which was not present in the multiple regression analysis (compare Figs. 3*B*, 2*C*), may be primarily explained by the awareness ratings not being controlled for when accuracy was entered into the regression model alone. Hence, most of the poststimulus covariance between EEG power and accuracy may be accounted for by the awareness ratings or processes related to both awareness and accuracy. However, even when awareness ratings were not controlled for in the accuracy measure, there was still no evidence for a significant relationship with prestimulus activity, providing further evidence that a relationship exists between prestimulus EEG power and visual awareness ratings which is independent of accuracy.

**Figure 4.**
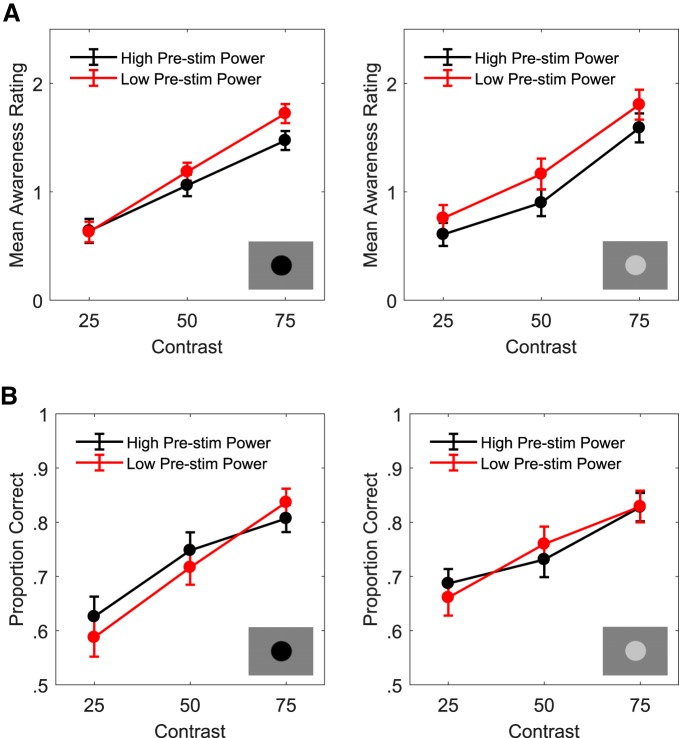
The inverse prestimulus power/PAS rating relationship is dependent on stimulus intensity. Group averaged PAS ratings (***A***) and proportion of correct responses (***B***) per above (black dots) and below (red dots) median power trials (single-trial power averaged over prestimulus portion of significant power/PAS cluster) are plotted as a function of stimulus contrast (25%, 50%, and 75% of detection threshold) separately for both luminance decrements (left column) and increments (right column). The analysis revealed that the inverse prestimulus power/subjective awareness relationship is not dependent on the luminance of the stimulus relative to the background (i.e., darker or lighter) but is dependent on the stimulus intensity, being present for higher but not for the lowest level of stimulus intensity. Again, no evidence for an effect of prestimulus power on accuracy was found. All error bars indicate within-subject ± SEM.

To further investigate the effect of prestimulus power (3–28 Hz) on perception, and any interaction this effect may have with stimulus features such as luminance or contrast, we performed an additional median power split ANOVA using the single-trial data from the prestimulus (−1:0 s) portion of the significant cluster. The proportion of correct responses and mean PAS rating were calculated separately for each luminance/contrast combination in each power bin (above and below median 3- to 28-Hz power). Figure 4 displays the group mean PAS ratings (Fig. 4*A*) and proportion of correct responses (Fig. 4*B*) per high power trials (black dots/lines) and low power trials (red dots/lines) at each contrast for both luminance decrements (left column) and increments (right column). A repeated measures ANOVA on the PAS ratings revealed a significant main effect of prestimulus power (*F*_(1,13)_ = 22.05, *p* < 0.001,$\eta_{p}^{2}$ = 0.629), a significant main effect of contrast (*F*_(2,26)_ = 86.357, *p* < 0.001,$\eta_{p}^{2}$ = 0.87, linear contrast: *F*_(1,13)_ = 167.482, *p* < 0.001,$\eta_{p}^{2}$ = 0.93), no significant main effect of luminance direction (*F*_(1,13)_ = 0.126, *p* = 0.728,$\eta_{p}^{2}$ = 0.01) and a significant prestimulus power × contrast interaction (*F*_(2,26)_ = 5.434, *p* = 0.011,$\eta_{p}^{2}$ = 0.295). *Post hoc* pairwise comparisons employed to explore the interaction term showed that PAS ratings (collapsed across luminance conditions) were significantly lower for high than for low power trials in the 75% (*t*_(13)_ = −4.314, *p* = 0.001, Cohen’s *d* = −1.169) and 50% (*t*_(13)_ = −4.179, *p* = 0.001, Cohen’s *d* = −1.164) contrasts but not for the 25% contrast (*t*_(13)_ = −0.1.959, *p* = 0.072, Cohen’s *d* = −0.547). No interaction between prestimulus power and luminance direction was detected (*F*_(1,13)_ = 2.558, *p* = 0.134,$\eta_{p}^{2}$ = 0.164). Hence, this analysis revealed that the inverse prestimulus power/subjective awareness relationship is not dependent on the luminance of the stimulus relative to the background (i.e., darker or lighter) but is dependent on the stimulus intensity, being present for higher levels but not for the lowest level of stimulus intensity.

A repeated measures ANOVA on the proportion of correct responses revealed a significant main effect of contrast (*F*_(2,26)_ = 50.683, *p* < 0.001,$\eta_{p}^{2}$ = 0.796, linear contrast: *F*_(1,13)_ = 114.459, *p* < 0.001,$\eta_{p}^{2}$ = 0.9) but no significant main effects of either prestimulus power (*F*_(1,13)_ = 0.309, *p* = 0.588,$\eta_{p}^{2}$ = 0.023) or luminance direction (*F*_(1,13)_ = 0.889, *p* = 0.363,$\eta_{p}^{2}$ = 0.064) as well as no significant interactions (all *p* > 0.05). Hence, no evidence for an effect of prestimulus power on accuracy was found, in line with the results of the regression analyses.

#### Catch trial analysis

**Figure 5.**
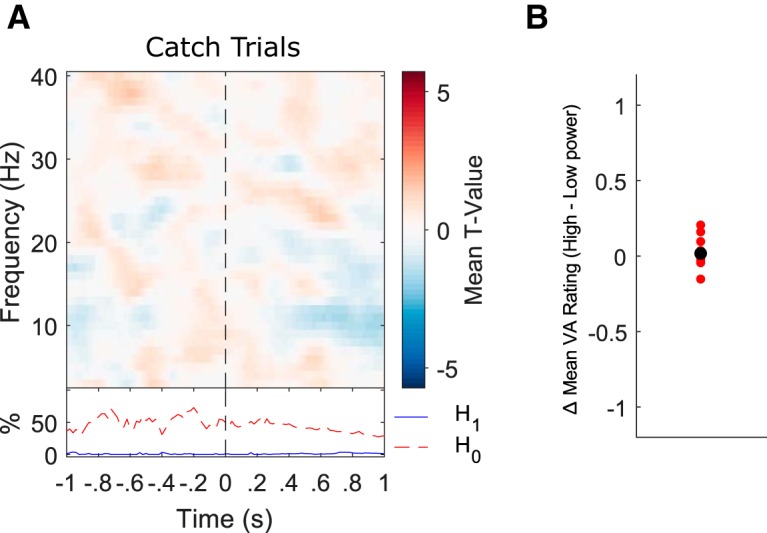
No relationship between oscillatory power and awareness ratings during catch trials. ***A***, The results of a single-trial regression analysis revealed that prestimulus power did not significantly covary with visual awareness ratings during catch trials. Stimulus onset is highlighted by a vertical black dashed line. The bottom inset plots the time course of the percentage of electrode-frequency points from the significant noncatch trial EEG/awareness cluster with BFs showing evidence for the null (H_0_: no EEG/catch trial PAS rating relationship; dashed red line) and alternative hypotheses, respectively (H_1_: significant EEG/catch trial PAS rating relationship; solid blue line). The percentage of data points providing evidence for H_0_ far outnumbers those providing evidence for H_1_. ***B***, Difference in mean PAS rating between above and below median power trials for each participant (black dot represents the mean difference value).

Figure 5*A* plots *t* values (averaged across all 60 electrodes) representing group-level tests of whether regression coefficients (EEG power vs catch trial PAS rating) from the individual single-trial analyses show a systematic linear relationship across participants. No significant relationship was found between EEG power and PAS ratings on catch trials in the time-frequency range examined in the cluster-based regression analysis. The percentage of data points providing evidence for H_0_ far outnumbered those providing evidence for H_1_ (Fig. 5*A*, bottom inset). The difference in catch trial PAS ratings between the high and low power bins of the prestimulus (-1:0 s) portion of the significant EEG power/awareness cluster are depicted in Figure 5*B*. The mean PAS ratings were 0.1392 for the high power catch trials and 0.1271 for the low power catch trials, respectively. A paired-samples *t* test revealed no significant difference in PAS ratings between high and low power catch trials (*t*_(13)_ = 0.5097, *p* = 0.6188, Cohen’s *d* = 0.158). Overall, no evidence was found for prestimulus EEG power influencing visual awareness ratings during catch trials.

#### Prestimulus phase does not predict visual awareness ratings or discrimination accuracy

**Figure 6.**
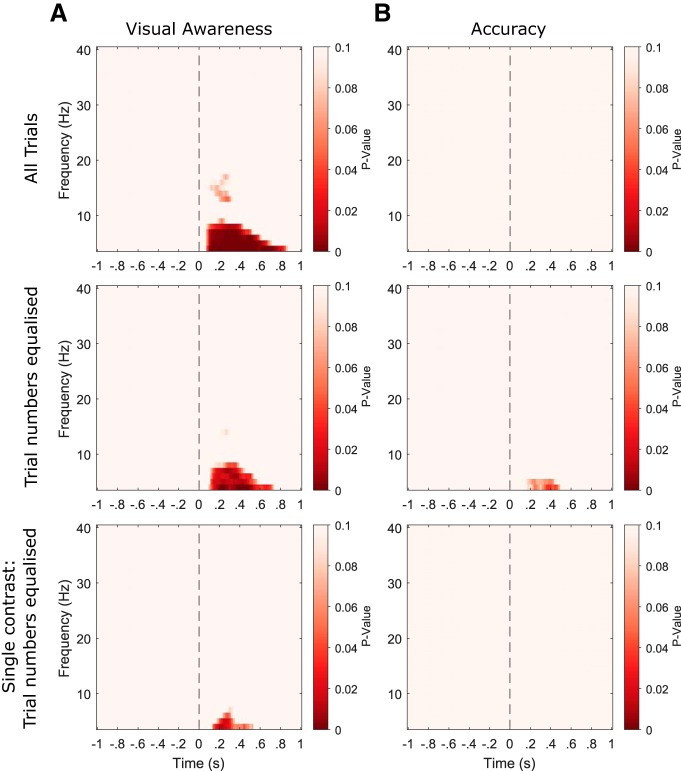
No relationship between prestimulus oscillatory phase and perception. ***A***, Time-frequency maps of *p* values (averaged over all participants and electrodes) from the visual awareness POS analyses. These values represent group-level tests of whether high (2 and 3) versus low (0 and 1) PAS rating trials tend to be phase locked to different (and hence preferred) phase angles. Stimulus onset is highlighted by a vertical black dashed line. ***B***, *p* values corresponding to the accuracy POS analyses. Both the visual awareness and accuracy analyses were performed on data with all trials included for each participant (top row), data where the trial numbers for each outcome were equalized within participants (middle row) and data from only the contrast level with the most even outcome split within participants (bottom row). No significant differences in prestimulus phase angle were found between high and low awareness rating trials, nor between correct and incorrect trials. Note that the statistical analysis was always restricted to prestimulus time points.

Figure 6 plots time-frequency maps of *p* values (averaged over all electrodes) from the phase opposition sum (POS) analysis. These values represent group-level tests of whether high (2 and 3) versus low (0 and 1) PAS rating trials (Fig. 6*A*) or correct versus incorrect trials (Fig. 6*B*) tend to be phase locked to different (and hence preferred) phase angles. The statistical analysis was restricted to prestimulus time points and no *p* values survived multiple comparison correction for either measure. This was the case for the analyses with all trials included (top row) and remained true also for the analyses in which relative trial numbers were equated between the two outcomes (middle row) and when only data from the contrast with the most even perceptual outcome split were included (bottom row). Hence, we found no evidence that prestimulus phase predicted either visual awareness ratings or discrimination accuracy.

## Discussion

We investigated the effects of prestimulus oscillatory activity on both discrimination accuracy and perceptual awareness ratings during performance of a 2-AFC luminance discrimination task. Single-trial regression analysis revealed an inverse relationship between prestimulus power (∼3-28 Hz) and subjective awareness ratings, but no relationship between prestimulus power and discrimination accuracy. Additionally, a phase opposition analysis found no evidence for a relationship between prestimulus phase and either subjective awareness ratings or discrimination accuracy. The results provide insights as to the processes by which prestimulus oscillatory activity influences perception and also highlight a neural dissociation between prestimulus predictors of subjective awareness and objective performance.

### Prestimulus EEG power predictors of perception

Prestimulus oscillatory activity in the α-band has repeatedly been shown to predict perceptual outcome (Ergenoglu et al., 2004; Thut et al., 2006; Van Dijk et al., 2008; Busch et al., 2009; Thut et al., 2012; Capilla et al., 2014; Limbach and Corballis, 2016; Iemi et al., 2017). Emerging evidence from studies employing psychophysical modeling techniques suggests that prestimulus α power primarily biases perception by influencing the decision criterion and not perceptual sensitivity (Lange et al., 2013; Chaumon and Busch, 2014; Limbach and Corballis, 2016; Sherman et al., 2016; Craddock et al., 2017; Iemi et al., 2017; Samaha et al., 2017). Iemi et al. (2017) formally tested two different models of α power on perception, namely a “baseline” model in which α power influences perception via modulation of baseline neural excitability and a “precision” model in which α power influences the precision of neural responses to task relevant stimuli. Using a combination of detection and discrimination tasks with perithreshold stimuli across two experiments, Iemi et al. (2017) found strong evidence in support of the baseline model by which decreased prestimulus α power (indexing high baseline neural excitability; Romei et al., 2008; Haegens et al., 2011; Lange et al., 2013) results in a more liberal criterion for detecting the presence of a target (regardless of whether this perception is veridical or not), with no change in discrimination sensitivity. Our data are congruent with this, suggesting that prestimulus power influences subjective awareness of stimuli while not necessarily influencing the ability of observers to discriminate task-relevant stimulus features. A baseline excitability dependent change in response bias may arise either at the nonsensory levels of decision strategy and metacognition or at the level of perceptual experience and stimulus visibility. We argue that our results are in line with the latter interpretation. We found no effect of EEG power on subjective awareness ratings during catch trials (i.e., when no stimulus was presented). Furthermore, the negative relationship between prestimulus power and awareness was only present for the strongest stimulus intensities. Hence, the relationship depended on there being a stimulus presented and how strong this stimulus was. This finding is not in line with a simple change in decision criterion but rather a response gain of stimulus visibility (see Chaumon and Busch, 2014 for a similar finding). Interestingly, Iemi and Busch (2017) recently employed a two-interval forced choice (2IFC) task and also found that the effect of prestimulus power on psychophysical performance is likely to represent a change in perceptual experience rather than a change in the decision strategy alone. It appears that the sensory information required to make discrimination judgments is not modulated by prestimulus power, whereas the level to which the stimulus reaches conscious awareness depends on baseline neural excitability.

Our results are also in line with those of Samaha et al. (2017), who found that prestimulus power was negatively correlated with decision confidence in a 2-AFC orientation discrimination task but was not correlated with decision accuracy. Although decision confidence and visual awareness ratings can sometimes be partially dissociated (Ramsøy and Overgaard, 2004; Zehetleitner and Rausch, 2013; Jachs et al., 2015; Rausch and Zehetleitner, 2016), they are likely to be correlated for many tasks in participants with intact visual systems. Hence, a parsimonious explanation for the results of Samaha et al. (2017) and the current study, also in line with the SDT studies discussed above, is that prestimulus power affects subjective measures of psychophysical performance (i.e., awareness and confidence ratings) but does not affect objective measures (i.e., decision accuracy). This somewhat counter-intuitive effect is congruent with a recently proposed Bayesian heuristic framework for decision confidence generation (Maniscalco et al., 2016; see also Zylberberg et al., 2012 and Fleming and Daw, 2017) in which the relative separation between the distributions of evidence in favor of each response in a 2-AFC task (i.e., patch darker or lighter in the current experiment) can remain stable while both distributions are shifted on a second dimension which determines the subjective awareness of the stimulus and confidence in the decision. Under this model, dissociation is possible between perceptual awareness/confidence and decision accuracy, which can account for observed suboptimal subjective measures of performance in perceptual discrimination tasks (Lau and Passingham, 2006; Rahnev et al., 2012; Zylberberg et al., 2014; Maniscalco and Lau, 2016; Maniscalco et al., 2016; Fleming and Daw, 2017). We propose that prestimulus power may primarily influence this second dimension (absolute awareness/evidence) and hence represent a predictor of the level of subjective awareness of an upcoming stimulus dissociable from neural predictors of objective performance.

### Prestimulus power predicting visual awareness: a specific α-band phenomenon?

Although the relationship we observed here between prestimulus power and visual awareness ratings was strongest in the classical α-band (∼8-12 Hz), it was widespread both topographically and in terms of frequency, spanning a wide range from 3-28 Hz. This is in line with previous studies on the effects of prestimulus power on perception which have also found negative relationships centered on, but not restricted to, the α-band (Limbach and Corballis, 2016; Iemi et al., 2017; Samaha et al., 2017). The broad-band nature of the effect, along with the fact that it was observed over almost the entire scalp (i.e., it is spatially nonspecific), may further suggest that it indexes fluctuations in neural excitability (Becker et al., 2011; Haegens et al., 2011; Becker et al., 2015; Iemi et al., 2017) and/or a global preparatory effect related to attention and conscious perception (He and Raichle, 2010), rather than a functionally relevant “oscillator” restricted to a narrow frequency band. This interpretation may also explain the lack of a relationship between oscillatory phase and perception because phase effects would be expected to be more tightly linked to an oscillation at a specific frequency.

### Prestimulus power influencing visual awareness rather than accuracy: a generalizable finding?

It should be noted that in the present study, the stimulus always appeared on the same (right) side of the screen and hence was entirely spatially predictable. In situations where attention is endogenously oriented to a certain spatial location (e.g., left or right hemifield) but there is some uncertainty about where the stimulus will appear, ipsilateral reduction/contralateral enhancement in cortical excitability (indexed by increase/decrease in α power, respectively) results in processing that is biased in favor of one hemifield versus the other (increasing detection rates and/or reducing reaction times for stimuli at attended positions; Worden et al., 2000; Sauseng et al., 2005; Kelly et al., 2006; Thut et al., 2006; Rihs et al., 2007; Foxe and Snyder, 2011). Converging evidence suggests that this effect represents a top-down control process (Capotosto et al., 2009; Marshall et al., 2015; van Diepen et al., 2016) that only occurs when irrelevant spatial regions actively compete with relevant spatial regions for limited attentional resources (Jensen and Mazaheri, 2010; Slagter et al., 2016), as is the case for probabilistic cueing tasks. A potentially interesting line of future research would be to test whether the predictions of the baseline model (i.e., α influencing criterion and not sensitivity; Iemi et al., 2017) also hold for stimuli presented at cued spatial locations.

It is also important to note that we only tested here for linear relationships between our EEG and perceptual measures and hence we cannot rule out the possibility that a nonmonotonic relationship between prestimulus power and visual sensitivity may exist (Linkenkaer-Hansen et al., 2004; Rajagovindan and Ding, 2011; Snyder et al., 2015). Additionally, the relationship between prestimulus activity and perception is likely to depend on the context and specific task demands as well as the underlying neural sources/mechanisms modulating oscillatory power (Mo et al., 2011; Lundqvist et al., 2013). For example, recent studies provide evidence that prestimulus α power encodes biases of upcoming sensory decisions induced by top-down predictions (Mayer et al., 2016) and predicts serial dependence (De Lange et al., 2013) and long-term nonstationarity of psychophysical performance (Benwell et al., 2017). Hence, the existence and direction of relationships between prestimulus EEG power and visual perception may depend on the context and nature of the task being performed.

### No relationship between prestimulus phase and perception

We found no evidence that prestimulus oscillatory phase predicts either subjective awareness or decision accuracy. A phase opposition analysis did not indicate that different perceptual outcomes (“high” vs “low” awareness ratings and correct vs incorrect responses) were phase-locked to different phase angles, such as was the case for detection “hits” versus “misses” before stimulus onset in Busch et al. (2009; see also Mathewson et al., 2011; VanRullen et al., 2011). One characteristic of the task that may have precluded phase from influencing perception was the temporal predictability of stimulus onset (i.e., there was always the same interval between the onset of the fixation cross and the stimulus, as the warning tone always occurred one second before stimulus onset). This may have limited spontaneous differences in phase angle distributions from influencing perceptual outcomes. In contrast to α power, van Diepen et al. (2015) recently found evidence against top-down modulation of α phase as a mechanism for attentional selection using a series of cueing paradigms with temporally predictable targets (although see Samaha et al., 2015). We therefore conclude that if phase does influence visual awareness or discrimination accuracy of perithreshold stimuli, then the effect must be weak in comparison to the effect of power (in line with Busch et al., 2009; Chaumon and Busch, 2014; see also Milton and Pleydell-Pearce, 2016). Additionally, there is some evidence that phase only influences perception in certain states of α power and attentional focus (Mathewson et al., 2009; Kizuk and Mathewson, 2017) and that previously observed prestimulus phase/perception relationships may have been overemphasized due to contamination of the filtered signal by target-evoked phase differences (Brüers and VanRullen, 2017 ). Hence, it is conceivable that the proposal that prestimulus phase angles (measurable with EEG) index perceptual or attentional cycles (Varela et al., 1981; VanRullen and Koch, 2003; Schroeder and Lakatos, 2009; Jensen et al., 2012) applies only under particular conditions, rather than representing a generally observable mechanism present in spontaneous EEG activity in the absence of visual input.

## Conclusion

The results of the current study add to a growing body of evidence suggesting that prestimulus α power negatively correlates with subjective measures of perception but does not correlate with objective measures. This intriguing finding suggests a dissociation between neural predictors of conscious awareness and task performance. We found no evidence for an effect of prestimulus phase on perception and hence conclude that any such effect is not as strong/consistent as the effect of power.

## Synthesis

Reviewing Editor: Bradley Postle, University of Wisconsin Decisions are customarily a result of the Reviewing Editor and the peer reviewers coming together and discussing their recommendations until a consensus is reached. When revisions are invited, a fact-based synthesis statement explaining their decision and outlining what is needed to prepare a revision will be listed below. The following reviewer(s) agreed to reveal their identity: Niko Busch

Reviewer #1

# Summary 

This study investigates how the present brain state - indicated by the power of ongoing neural oscillations - influences the perception of upcoming stimuli. Specifically, the study elaborates on recent findings suggesting that pre-stimulus oscillations in the alpha band affect not the accuracy of visual perception, but the detection bias. Here, the authors tested whether this bias in turn affects observers' subjective perception in a signal detection task. Indeed, a state of pre-stimulus alpha power amplified subjects' subjective feeling of awareness, but not their objective accuracy. 

The study addresses a timely topic. The paper is well written; the study design and analysis are sound and the results largely justify the conclusions. I expect that the study will be met with widespread interest. 

I do not have much to criticize. I do have a few concerns and questions, but I expect that it will be rather easy for the authors to accommodate them. In fact, my comments are intended to be constructive, believing that this study has great potential. 

# Analysis of luminance increments and decrements 

Most previous studies on this issue have used stimuli that were defined as luminance \*increments\* (e.g. Ergenoglu, van Dijk, Chaumon, Iemi etc.). One interesting aspect of this study is that stimuli were either luminance increments or decrements. I would not actually say that your stimuli were defined by contrast; this would only be the case for a stimulus that has lighter and darker components, compared to the background, e.g. a checkerboard or grating stimulus. Nonetheless, as far as I know, nobody has tested yet whether pre-stimulus oscillations influence detection of luminance decrements in the same way as for increments. This is an interesting question because it is not immediately obvious whether stronger neural excitability/weak oscillations makes a dark patch subjectively even darker relative to the background (making it easier to see) or less dark relative to the background (making it more difficult to see). If it turns out that weak pre-stimulus oscillations increase awareness for both luminance increments and decrements, this would be a curious finding. It implies that weak oscillations/excitability amplify the sensation, and that the “direction” of amplification depends on stimulus luminance: luminance increments get brighter while luminance decrements get darker. However, how does the visual system know, without access to the external world, whether an external stimulus was an increment or decrement and in which direction it needs amplification? I think this paradigm offers a great potential for a more in depth analysis, to get a deeper understanding of these effects. 

First, I think it is necessary to analyze trials separately for luminance increments and decrements. This concerns the behavioral analysis as well as the EEG power analysis. Specifically, it is important to know whether pre-stimulus power affects awareness ratings in the same way for increments and decrements. This analysis could potentially be restricted to the significant cluster from your initial analysis. It would be interesting to see to a “psychometric function”, i.e. awareness ratings and accuracy as a function of luminance (across all six/seven levels), and compare these functions for trials with strong/weak pre-stimulus power (similar to Chaumon). In my opinion, such an analysis also puts dependent and independent variables in the correct order. While your regression analysis treats EEG power as a dependent variable, the causality is actually the other way around: pre-stimulus power causes (or is in turn correlated with the underlying cause) effects on stimulus perception. 

Second, it would be valuable to include stimulus-absent trials in the analysis. I understand that 2AFC accuracy is not defined for catch trials, but the awareness ratings can be analyzed. These trials correspond to the stimulus-absent trials in the recent studies by Limbach and Iemi, and one would expect that pre-stimulus power affects awareness in the same way. 

# Awareness vs confidence 

In the Introduction, you seem to suggest that this study addresses “the level of processing at which these effects occur”, i.e. whether pre-stimulus power modulates decision strategy or subjective awareness. The reasoning seems to be that, unlike confidence, the visual awareness scale is a pure measure of awareness. Thus, any effects on the VAS rating cannot reflect decision strategy. In my opinion, this reasoning takes at face value that subjects do not use the VAS scale as a proxy for confidence. How do we know that this was indeed not the case? And how were subjects instructed to use the VAS scale (and how not to use it)? Maybe it would even be valuable to report the instructions verbatim. 

Indeed, in the Discussion, you acknowledge that VAS ratings and confidence are often correlated. Moreover, it is not clear to me how you answer the question from the Introduction: is it clearly an effect on awareness, rather than a decision bias? If you find that the results are not conclusive regarding this question, it would be important to know that. 

# Regression analysis 

Effects of EEG power on awareness and accuracy were analyzed with a linear regression model with two regressors: VAS ratings and accuracy. I have several questions regarding this analysis: 

--Please explain how this regression handles data, in which regressors are correlated among each other. After all, the behavioral analysis shows that both awareness and accuracy increase with contrast, so we would expect them to be strongly correlated. You seem to assume that the results for each regressor are independent of the other regressor. Is this really the case? If so, the results should not change if you compute two separate models, one for each regressor. 

--I am surprised that accuracy is not related to power at any time, not even post-stimulus! I would have expected that accurate perception goes along with some kind of P3-like ERP, and this should be visible in the tf-plot. That is, it should be, unless all the covariance between accuracy and EEG power was already explained “away” by the awareness ratings. 

--Moreover, given that accuracy is strongly related to stimulus contrast, I would expect to see an effect for accuracy resembling a visual evoked potential: stronger contrast causes better accuracy, causing stronger ERP. 

--According to your statement in the Discussion that effects of power on perception “should be limited to situations in which the stimulus itself provides little sensory stimulation”, it would make sense to include contrast as a regressor (and an interaction term, too). 

# Minor 

--The results show that power is influenced by awareness (or the other way round) both in the pre-stimulus and post-stimulus time range. But the strongest effects appear post-stimulus. To what extent are the pre-stimulus results affected by post-stimulus signals due to the temporal smear of the time-frequency transform? 

--I wonder why stimuli were always presented at the same peripheral location. I suppose this makes it tempting for subjects to look at this location. The Methods only mention that “All epochs were then visually inspected and removed if contaminated by residual eye movements...”. How were eye movements identified, according to which criterion, and how sensitive was this procedure? And how was accurate fixation monitored? After all, if a subject already fixated on the stimulus location, rather than the fixation cross, at the beginning of the trial, this would not even be visible in the EOG. 

--The results include several interesting insignificant findings. It would be interesting to know whether these results indicate evidence for the null hypothesis or are merely inconclusive. I suggest complementing this analysis with a Bayesian analysis that directly quantifies the evidence for H1 or H0. Such measures (e.g. Bayes factors) are relatively easy to compute with standard software (e.g. R or JASP). 

--Discussion: “Another consequence of an interpretation of the effects of pre-stimulus alpha power on perception as representing fluctuations in cortical excitability is that they should be limited to situations in which the stimulus itself provides little sensory stimulation. This follows because in cases where the stimulus is suprathreshold and provides strong sensory stimulation, the influence of ongoing fluctuations in baseline excitability is unlikely to alter the perceptual outcome (perhaps with the exception of reaction time (Kelly and O'Connell, 2013, van den Berg et al., 2016)), whereas in cases of low sensory stimulation (with perithreshold stimuli) fluctuations in ongoing alpha power may push a stimulus in or out of awareness. Indeed, most experiments investigating the effects of prestimulus oscillations on perception have been restricted to tasks employing perithreshold stimuli.” - One exception is the study by Chaumon et al. who presented stimulus intensities ranging from very weak to clearly visible. The effect of pre-stimulus alpha power was strongest for high-intensity stimuli. This effect corresponds to a response gain effect, which is often seen e.g. in studies on involuntary attention or arousal. I think this should be mentioned here. Moreover, it would be interesting to see how your study compares to their finding (see my comment above). 

- The authors' choice of analyses is a little curious, as they use linear regression to predict prestimulus EEG activity from poststimulus behavior. Hence, they are in a way postdicting EEG activity. While the regression model of course does not care about the temporal relationship, predicting behavior from EEG using a logistic/multinomial regression would have perhaps been more intuitive. However, the current model also seems overly restrictive as it does not include an interaction term between accuracy and PAS; the authors should add this term and interpret their results in light of a possibly significant interaction. This could significantly change the interpretation of results. Furthermore, I found no treatment of multicolinearity in the manuscript. It seems very likely that the predictors are very highly correlated, and the authors should assure that they built a stable model with sufficient explanatory power, and - if warranted - use appropriate methods to disentangle accuracy and PAS predictors. In this context, it would be good to present an analysis of the relationship of PAS and accuracy for the behavioral data in general. Finally, the authors should be clear in the discussion that they are merely assessing linear relationships with their analyses, and that non-linear relationships are alternative explanations for their null results. 

- The entry level threshold for cluster correction for multiple comparisons is set at 0.05, which is extremely liberal and does not necessarily guarantee the expected control over false positives (Eklund, Nichols, Knutsson 2016). Where FDR was used, the q-value should be given to assess the percentage of allowed false positives. 

Reviewer #2

In the manuscript entitled “Prestimulus EEG power predicts conscious awareness but not objective visual performance”, the authors present results from an EEG study in which subjects performed a luminance discrimination task, and also rated their subjective experience of the visual stimuli on a trial-by-trial basis. The authors assess prestimulus baseline activity and find that power in the 3-28 Hz range correlates with subjective experience, but not discrimination performance, and furthermore, that oscillatory phase correlates with neither behavioral measure. This paper is one of a series of papers assessing the influence of prestimulus activity on perception, and investigates an interesting question, namely a potential dissociation between objective performance and subjective awareness, which still lacks a definitive answer. 

- The authors' choice of analyses is a little curious, as they use linear regression to predict prestimulus EEG activity from poststimulus behavior. Hence, they are in a way postdicting EEG activity. While the regression model of course does not care about the temporal relationship, predicting behavior from EEG using a logistic/multinomial regression would have perhaps been more intuitive. However, the current model also seems overly restrictive as it does not include an interaction term between accuracy and PAS; the authors should add this term and interpret their results in light of a possibly significant interaction. This could significantly change the interpretation of results. Furthermore, I found no treatment of multicolinearity in the manuscript. It seems very likely that the predictors are very highly correlated, and the authors should assure that they built a stable model with sufficient explanatory power, and - if warranted - use appropriate methods to disentangle accuracy and PAS predictors. In this context, it would be good to present an analysis of the relationship of PAS and accuracy for the behavioral data in general. Finally, the authors should be clear in the discussion that they are merely assessing linear relationships with their analyses, and that non-linear relationships are alternative explanations for their null results. 

- The entry level threshold for cluster correction for multiple comparisons is set at 0.05, which is extremely liberal and does not necessarily guarantee the expected control over false positives (Eklund, Nichols, Knutsson 2016). Where FDR was used, the q-value should be given to assess the percentage of allowed false positives. 

- The authors focus in their figures and much of their discussion on prestimulus alpha power. However, their effects seem to span a range from 3-28 Hz, which includes the delta, theta, and beta bands. Furthermore, the effect is spread over the entire scalp and does not exhibit lateralization. It thus seems much more likely that the authors are investigation a global effect, perhaps a slow cortical potential, which, coincidentally, has also been related to conscious perception (He & Raichle, 2010). I strongly recommend that the authors either provide an analysis that supports their focus on alpha or rewrite their manuscript in light of the broadband activity they observed. Relatedly, the presentation of previous results on prestimulus alpha band activity seems biased towards studies that support a negative relationship between alpha power and perception, while studies reporting the opposite (e.g., Linkenkaer-Hansen et al. 2004, Babiloni et al. 2006, Becker et al. 2011, Mo et al.2011, Mayer et al. 2016) and models explaining bidirectional effects of alpha on perception (Lundqvist et al. 2013) are entirely ignored. They should be included into the introduction and discussion. 

- More details on the filters should be provided (type, order, etc.). 

- page 19: pre-stimulus activity cannot logically be a NCC; hence, the fact that alpha power may constitute a NCC-pr does not “follow” from the arguments in the preceding paragraph. 

- On page 3, I suggest replacing “candidate mechanism” by “possible explanation; on page 17, I suggest replacing “mechanisms” by “processes”.
